# On the edge of awareness: ictal consciousness and psychosis in focal epilepsy

**DOI:** 10.1192/j.eurpsy.2026.10511

**Published:** 2026-07-31

**Authors:** A. Tarrada, S. Baltassis

**Affiliations:** 1 Unit of neuropsychiatry, Psychotherapeutic Center of Nancy, Nancy; 2 Neurology, Hospital of Pitie-Salpêtrière, Paris, France

## Abstract

**Introduction:**

Psychoses of epilepsy (POE) are psychotic disorders arising after epilepsy onset, affecting up to 13% of patients with focal epilepsy. Based on their temporal relationship with seizures, POE is classified as peri-ictal psychosis —with postictal psychosis (PIP) being most common—or interictal psychosis. These subtypes often overlap within the same patients, suggesting shared mechanisms.

**Objectives:**

While interictal risk factors are well documented, ictal correlates remain poorly characterized. This study assessed the association between objective ictal features, ictal consciousness and POE.

**Methods:**

We included 112 patients with drug-resistant focal epilepsy who underwent epilepsy-specific psychiatric assessment and long-term video-EEG or stereo-EEG (S-EEG) monitoring. Seizure semiology and epilepsy features were compared between patients with (n = 15) and without POE (n = 97). Ictal consciousness was assessed using the Consciousness Seizure Scale(CSS). For analysis, CSS scores were categorized into three levels: 0–1 (preserved consciousness), 2–6 (altered), and 7–9 (abolished). Seizure onset zones were examined in S-EEG patients (n = 37).

**Results:**

POE was associated with altered ictal consciousness (p = .04), following a non-linear pattern peaking at intermediate CSS scores (p = .015). POE patients were older (p = .005), had longer epilepsy duration (p= .005), and higher depression (p= .002) and anxiety scores (p =.001). Non-significant trends were found for lateral epilepsy (p = .07) and learning disabilities (p= .06).

**Image 1:**
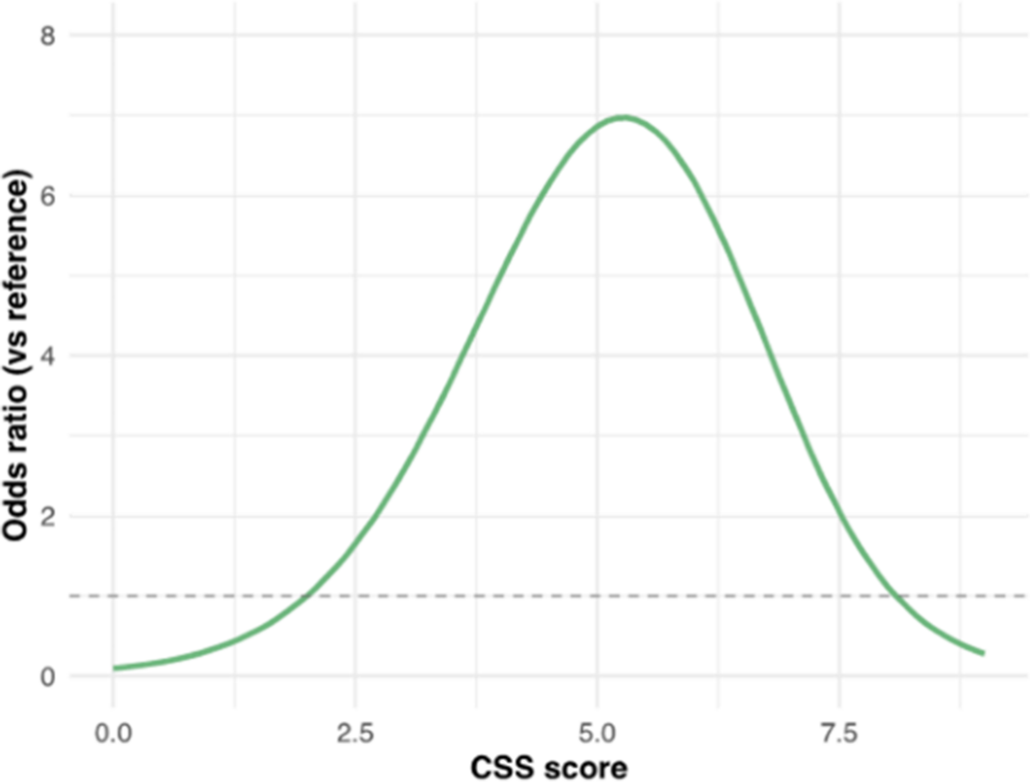
Image 1: Long description.

**Conclusions:**

POE was associated with intermediate ictal impairement of consciousness, possibly reflecting partial disconnection from reality. These patients also had longer disease duration, possibly reflecting a cumulative seizure burden. Lateral temporal involvement—implicated in impaired awareness and psychiatric vulnerability—raises whether psychosis risk stems from seizure focus or ictal consciousness quality.

**Disclosure of Interest:**

None Declared

## Long descriptions

### Image 1: Long description

The graph uses a Cartesian coordinate system with a light gray grid.

* The horizontal X-axis is labeled C S S score with numerical markers at 0.0, 2.5, 5.0, and 7.5.

* The vertical Y-axis is labeled Odds ratio (vs reference) with numerical markers at 0, 2, 4, 6, and 8.

* A horizontal dashed gray line represents the reference baseline at Y equals 1.

* A solid green curve represents the data. It begins near 0 on the Y-axis at an X-score of 0.0.

* The curve rises steadily, crossing the reference line at a C S S score of approximately 2.0.

* It reaches a peak Odds ratio of approximately 7.0 at a C S S score of roughly 5.3.

* After the peak, the curve descends symmetrically, crossing back below the reference line at a C S S score of approximately 8.5, ending near 0.3 on the Y-axis.

Navigate back to Image 1:.

